# Soy Isoflavones Protect Neuronal PC12 Cells against Hypoxic Damage through Nrf2 Activation and Suppression of p38 MAPK and AKT–mTOR Pathways

**DOI:** 10.3390/antiox11102037

**Published:** 2022-10-16

**Authors:** Yongzhu Zhang, Liqing Yin, Jiajia Dong, Xiudong Xia

**Affiliations:** 1 Key Research Laboratory of Chinese Medicine Processing of Jiangsu Province, College of Pharmacy, Nanjing University of Chinese Medicine, Nanjing 210023, China; 2 Institute of Agricultural Product Processing, Jiangsu Academy of Agricultural Sciences, Nanjing 210014, China; 3 Key Laboratory of Food Quality and Safety of Jiangsu Province-State Key Laboratory Cultivation Base of Ministry of Science and Technology, Laboratory of Quality and Safety Risk Assessment for Agro-Products of Ministry of Agriculture and Rural Affairs, Institute of Food Safety and Nutrition, Jiangsu Academy of Agricultural Sciences, Nanjing 210014, China

**Keywords:** phenolic compounds, neuroprotective effects, apoptosis, cell cycle, molecular docking analysis

## Abstract

Isoflavones are a class of major phenolic compounds, derived from soybeans, that possess unique therapeutic and biological properties. The possible mechanisms of isoflavone-mediated protection of neuronal PC12 cells against hypoxic damage was investigated in this study. Isoflavones showed potential neuroprotective effects by increasing cell viability, decreasing the level of reactive oxygen species (ROS), and inhibiting apoptosis and cell cycle arrest in cobalt chloride (CoCl_2_)-induced hypoxic damage. A Western blot analysis indicated that isoflavones decreased apoptosis by up-regulating the Bcl-xL protein and down-regulating the Bax protein. They further reduced the S-phase fraction of the cell cycle by down-regulating the p21 protein and up-regulating the cyclin A protein levels. Additionally, isoflavones activated Nrf2 protein translocation and inhibited the p38 MAPK and AKT–mTOR pathways. A molecular docking analysis further revealed that isoflavones displayed a potential competitive interaction with the Nrf2 protein for Keap1. Our findings suggest that isoflavones could be a potent neuroprotective phytochemical in soybeans and their products.

## 1. Introduction

Brain hypoxia, one of the critical complications caused by impaired brain metabolism, induces a series of pathological responses in brain functions [1]. Compared with other types of cells, the neuron is more sensitive to altered oxygen levels, especially hypoxia. For instance, the central nervous system was found to suffer a severe functional impairment after blocking the oxygen supply to the brain for 5 s [2]. Functional and structural injuries were observed in central neurons when chronic intermittent hypoxia occurred in obstructive sleep apnea [3]. Some estimates have demonstrated that almost half of all neonatal encephalopathies might be attributed to hypoxia–ischemia [4]. Hypoxia also increases the risk of neurodegenerative disorders, including Parkinson’s and Alzheimer’s diseases [1].

In recent years, some natural bioactive compounds have been found to be effective in reducing hypoxia-induced brain injury. Resveratrol improved the synaptic plasticity in hypoxic–ischemic brain injury in neonatal mice by decreasing silent information regulator 1 (SIRT1)/nuclear factor kappa-B (NF-κB) signaling-mediated neuroinflammation [5]. Mangiferin attenuated cerebral hypoxia/reoxygenation injury in neuroblastoma cells by activating the SIRT1/peroxisome proliferator-activated receptor-γ coactivator 1α (PGC-1α) signal [6]. Curcumin protected SH-SY5Y cells against hypoxia-reoxygenation injury by ameliorating apoptosis and oxidative stress through improving the apurinic/apyrimidinic endonuclease 1 (APE1) levels and activating phosphatidylinositol 3-kinase/protein kinase B (PI3K/AKT) pathway [7]. Soybean isoflavones (SIs) are the main phenolic compounds in soybeans and their products. They have gained increasing attention due to their unique therapeutic and biological properties with good availability, edibility, and biodegradability. SIs have been reported to exhibit obvious inhibitory effects on neuronal apoptosis induced by a noxious stimulus in vivo [8]. Genistein can ameliorate amyloid beta (Aβ)25-35-induced neuronal apoptosis by modulating estrogen receptors, choline acetyltransferase, and glutamate receptors [9]. In addition, SIs such as daidzein and genistein could directly cross the blood–brain barrier into the brain to exert physiological functions [10]. However, data on the neuroprotective effect of SIs on a hypoxia-induced injury are not available.

Oxidative stress plays a vital role in hypoxia-induced neuronal apoptosis [1]. Nuclear factor erythroid 2-related factor 2 (Nrf2) pathway activation significantly enhances cell survival under oxidative stress [11]. Autophagy, as a crucial cell survival mechanism, has been reported to be deeply involved in mitochondrial reactive oxygen species (ROS) production and neuronal apoptosis in a hypoxic injury, which is directly regulated by the Akt-mechanistic target of the rapamycin (mTOR) pathway [12,13]. Additionally, cell apoptosis occurs after the p38 mitogen-activated protein kinase (p38 MAPK)-dependent activation of pro-apoptotic proteins followed by the release of cytochrome c, consequently inducing a caspase cascade [14]. Therefore, targeting these pathways is a viable strategy to evaluate the neuroprotective effect of SIs against hypoxic injury. In this study, cobalt chloride (CoCl_2_)-treated PC12 cells were used as a hypoxia-injured model in vitro to evaluate the potential neuroprotection of SIs. The effects of SIs on the ROS production, cell apoptosis, and the cell cycle were also investigated. Furthermore, the effects of SIs on the Nrf2, p38 MAPK, and Akt–mTOR pathways were studied to explore the underlying protective mechanism of nerve cells. The purpose of the present study was to characterize the neuroprotective effects of SIs on hypoxic injury and discuss the underlying molecular mechanisms.

## 2. Materials and Methods

### 2.1. Materials

An SI mixture containing 51.81% daidzin, 19% glycitin, 7.94% genistin, 2.45% daidzein, 0.81% glycitein, and 0.52% genistein, and their standards, were obtained from Shanghai Yuanye Bio-Technology Co., Ltd. (Shanghai, China) [15]. Penicillin/streptomycin, fetal bovine serum (FBS), and Dulbecco’s modified Eagle medium (DMEM) were purchased from Gibco (CA, USA). PC12 cell lines were purchased from Nanjing Keygen Biotech. Co., Ltd. (Nanjing, China). The kits for ROS, apoptosis, the cell cycle, and the protein content were obtained from the Shanghai Beyotime Institute of Biotechnology (Shanghai, China). Antibodies for β-tubulin, Bax, cyclin A, p21, Bcl-xL, hypoxia-inducible factor-1 alpha (HIF-1α), LC3, p62, Nrf2, p-Akt, Akt, p-mTOR, mTOR, p-p38 MAPK, p38 MAPK, and LamB were obtained from Shanghai Abmart Inc. (Shanghai, China).

### 2.2. Cytotoxicity

Cell viability was determined using an MTT assay [16]. In brief, PC12 cells were inoculated in a culture medium consisting of 90% DMEM, 10% FBS, 100 U/mL of penicillin, and 100 μg/mL of streptomycin for at least 2 days. Then, cells were transferred to a 96-well plate with 1 × 10^4^ cells/well and cultivated for 12 h. Subsequently, various concentrations of CoCl_2_ or SIs were added to treat cells for another 12 h. Then, a prepared MTT solution was added and the plate was maintained under the same conditions for 4 h. After incubation, the medium was discarded and 150 μL of DMSO was pipetted into each well. Then, the plate was mildly shocked for 10 min. The absorbance was read at 490 nm.

### 2.3. Influence of SIs on Cell Viability Loss

A concentration of 1 × 10^4^ cells/well was inoculated in a 96-well plate for 12 h. CoCl_2_ (800 μM) was used to establish the hypoxic-injured model of PC12 cells. Various doses of SIs (25–75 μg/mL) were added to study their protective effects. Cell viability was determined using the MTT assay as described above.

### 2.4. Influence of SIs on ROS Production

A concentration of 2 × 10^5^ cells/well was inoculated in a 6-well plate for 12 h. Cells were then stimulated with 800 μM CoCl_2_ and treated with or without SIs for another 12 h. An ROS assay kit was used to determine the ROS level according to its instruction. In brief, PC12 cells were collected using trypsin and exposed to dichlorofluorescin diacetate (DCFH-DA, 10 μM) for 20 min. After centrifugation, cells were suspended in a serum-free medium. The fluorescence was quantified using a flow cytometer (Accuri C6 Plus, BD, New York, USA).

### 2.5. Influence of SIs on Cell Cycle

Cells were treated with CoCl_2_ and SIs as described above and collected using trypsin. A cell cycle kit was used to measure the cell cycle state of PC12 according to its instruction. Briefly, PC12 cells were washed using cold PBS and then fixed using ethanol (70%, *v*/*v*) for 0.5 h. Subsequently, cells were exposed to 0.5 mL of a propidium iodide (PI) staining reagent in the dark at 37 °C for 0.5 h. The fluorescence was detected using a flow cytometer. The cell apoptosis-related proteins, p21 and cyclin A, were analyzed using a Western blot.

### 2.6. Influence of SIs on Cell Apoptosis

Cells were treated with CoCl_2_ and SIs as described above and collected using trypsin. A cell apoptosis kit was used to evaluate the PC12 cell apoptosis according to the manufacturer’s instructions. Briefly, 0.5–1.0 × 10^5^ cells were suspended in 195 μL of annexin V–fluorescein isothiocyanate (FITC) binding buffer. Then, the mixture was mixed with V–FITC (5 μL), followed by PI (10 μL) for 20 min. The fluorescence was detected using a flow cytometer. The cell apoptosis-related proteins, Bax and Bcl-xL, were analyzed using a Western blot.

### 2.7. Western Blot Analysis

A concentration of 2 × 10^5^ cells/well was inoculated in a 6-well plate for 12 h. Then, cells were treated with SIs or isoflavone monomers for another 12 h. Subsequently, an RIPA buffer was used to lyse the cells. The supernatant was retained after centrifugation. The protein concentration was quantified based on the BCA method. The sample containing proteins (30 µg/lane) was loaded on a sodium dodecyl sulfate-polyacrylamide gel. Subsequently, protein bands were transferred onto a polyvinylidene fluoride membrane and exposed to antibodies against HIF-1α, LC3, p62, Nrf2, p-Akt, Akt, p-mTOR, mTOR, p-p38 MAPK, p38 MAPK, LamB, and β-tubulin. Immunoblot images were obtained using a detection system (ImageQuant LAS4000mini, GE, Boston, USA). Protein densitometry was determined using ImageJ software.

### 2.8. Model Assessment and Molecular Docking

A molecular docking analysis was conducted according to the methods in our previous report [15]. Briefly, the Keap1 protein (PDB ID: 4l7b) was found in the protein data bank (PDB). The Discovery Studio program was used to analyze the 3D molecular structure. All water molecules and other chains were removed from the Keap1 protein to evaluate the conformation more accurately. The 3D structures of six isoflavone monomers were drawn using ChemBioOffice 2018 software, as shown in our previous study [15]. All compounds were geometrically optimized by CHARMm in Discovery Studio. The interactions between isoflavone monomers and Keap1 were visualized using LigPlot+ and PyMol molecular graphic systems.

### 2.9. Statistical Analysis

All experiments were repeated at least three times. Data were reported as a mean and standard deviation (SD). Statistical analyses were carried out using SPSS software and a variance analysis was carried out using one-way ANOVA followed by Duncan’s post hoc multiple comparisons test. A value of *p* < 0.05 was considered significant in this study.

## 3. Results

### 3.1. Cytotoxicity and Inhibition of Isoflavones on Cell Viability Loss

**Figure 1:**
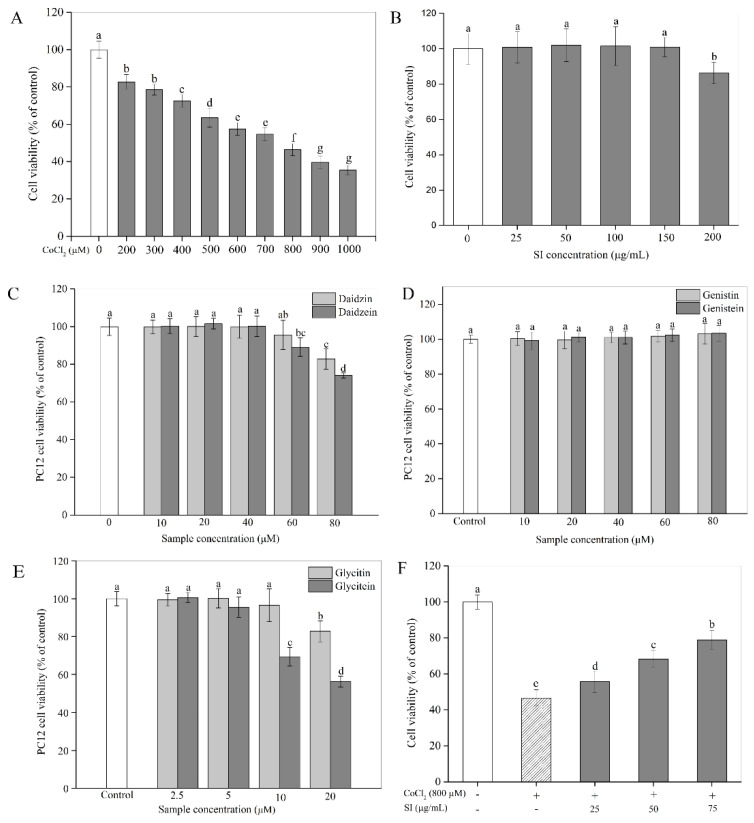
Cytotoxicity and the protective effect of SIs on CoCl_2_-induced cell viability loss. (**A**) Cytotoxicity of CoCl_2_. (**B**) Cytotoxicity of SIs. (**C**) Cytotoxicity of daidzin and daidzein. (**D**) Cytotoxicity of genistin and genistein. (**E**) Cytotoxicity of glycitin and glycitein. (**F**) The protective effect of SIs on CoCl_2_-induced cell viability loss. Means with different small letters were significantly different (*p* < 0.05).

In the present study, the in vitro hypoxia-injured model of the PC12 cells was established using CoCl_2_. To determine the optimum treatment dose, PC12 cells were stimulated with various concentrations of CoCl_2_ ranging from 200 to 1000 μM for 12 h. As shown in Figure 1A, the results of the MTT assay revealed that exposure to CoCl_2_ for 12 h induced significant (*p* < 0.05) cytotoxicity in a concentration-dependent way. The viability of the PC12 cells exposed to 800 μM CoCl_2_ was only about 46% of the control value. Hence, a CoCl_2_ concentration of 800 μM was used to simulate hypoxic conditions in the follow-up experiments. The cytotoxicity of SIs and isoflavone monomers was also investigated in this study. From Figure 1B, an SI treatment for 12 h exhibited no toxic effect on cell viability at concentrations from 25 μg/mL to 150 μg/mL, indicating that the safe doses of SIs were below 150 μg/mL for PC12 cells. The daidzin, daidzein, glycitin, and glycitein treatments for 12 h were non-toxic to PC12 cells below 60 μM, 40 μM, 10 μM, and 5 μM, respectively, indicating that the cytotoxicity of aglycone isoflavones was stronger than their corresponding glycosides (Figure 1C,E), whereas genistin and genistein showed no cytotoxicity to PC12 cells even at a high dose of 80 μM, demonstrating that the cytotoxicity of genistin and genistein is much weaker than the other monomers (Figure 1D). Isoflavone monomers at safe doses were used in the following study.

**Figure 2:**
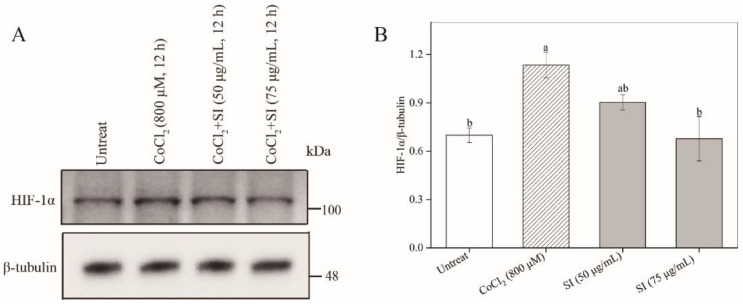
The effects of CoCl_2_ and SIs on the expression level of HIF-1α. (**A**) Western blot analysis of HIF-1α protein. (**B**) The relative expression level of HIF-1α protein. Means with different small letters were significantly different (*p* < 0.05).

To study the protective potency of SIs, we first examined their effects on PC12 cell viability under a hypoxic injury. As shown in Figure 1F, a hypoxic injury significantly inhibited PC12 cell proliferation compared with the untreated control. However, this unfavorable situation was effectively improved by SIs. The SI treatment at a dose from 25 μg/mL to 75 μg/mL for 12 h rescued the cell viability loss induced by CoCl_2_ in a concentration-dependent way. For example, the cell viability significantly (*p* < 0.05) increased by 68.58% of that in the hypoxia-injured group after the SI treatment at 75 μg/mL. Similar results were also observed for six isoflavone monomers (Figure S1). Additionally, to further confirm the success of a hypoxia injury model induced by CoCl_2_ and the protective effect of SIs against hypoxic injury, the expression level of HIF-1α was analyzed. As shown in Figure 2A,B, a CoCl_2_ treatment for 12 h significantly increased the HIF-1α protein level in PC12 cells. Considering the significant cell viability loss induced by CoCl_2_, the model of sustained hypoxia was successfully established by CoCl_2_ in this work. However, the SI treatment significantly decreased the HIF-1α expression and increased the cell viability under hypoxic injury (Figure 1F), indicating that soy isoflavones acted against hypoxic injury despite sustained hypoxia.

### 3.2. Attenuation of ROS Production by SIs

**Figure 3:**
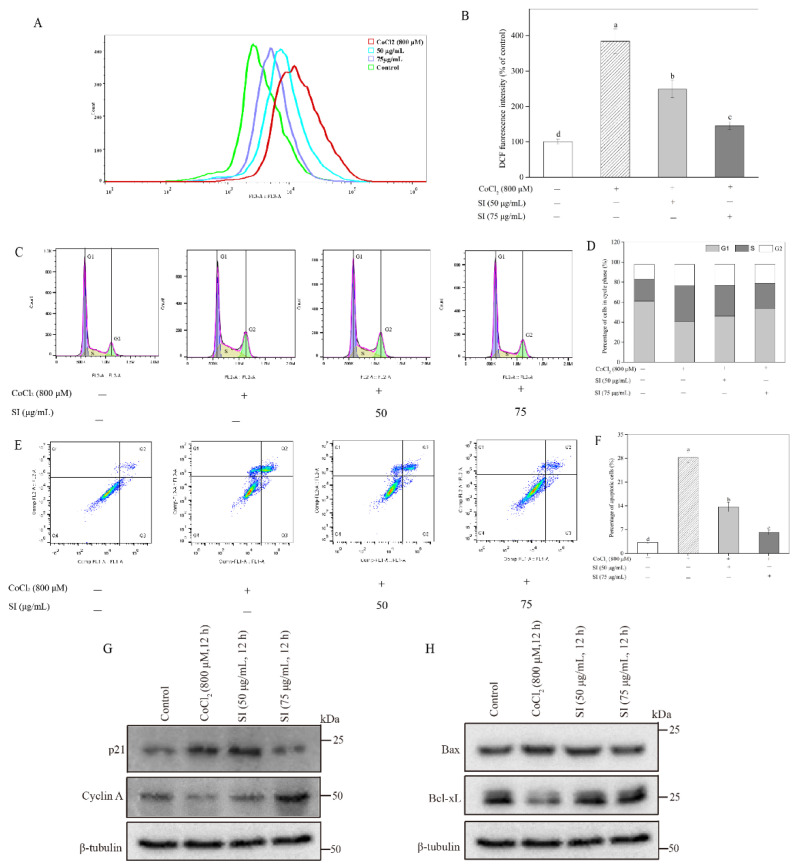
Effects of SI on ROS production, cell cycle arrest, and apoptosis induced by CoCl_2_. (**A**) ROS levels analyzed by flow cytometer. (**B**) Fluorescence intensity in (**A**). (**C**) Cell cycle analyzed by flow cytometer. (**D**) Cell cycle in (**C**). (**E**) Cell apoptosis analyzed using flow cytometer. (**F**) Cell apoptosis rate in (**E**). (**G**) Western blot analysis of p21 and cyclin A proteins. (**H**) Western blot analysis of Bax and Bcl-xL proteins. Means with different small letters were significantly different (*p* < 0.05).

As shown in Figure 3A,B, the CoCl_2_ treatment significantly increased the ROS level in PC12 cells, which was 3.84 times that in untreated cells, indicating that severe oxidative stress occurred in the PC12 cells after the CoCl_2_ stimulation. However, the SI treatment at 50 μg/mL and 75 μg/mL significantly (*p* < 0.05) attenuated the CoCl_2_-induced ROS production. Notably, the ROS level in the 75 μg/mL SI-treated cells significantly (*p* < 0.05) decreased by 62.06% compared with that in only CoCl_2_-treated PC12 cells, suggesting that SIs are potential agents against oxidative stress in hypoxic injury.

### 3.3. Improvement of Cell Cycle by SIs

As shown in Figure 3C,D, the S fraction of the cell cycle exhibited a significant increase after the CoCl_2_ exposure, which increased by 63.22% compared with the untreated control. Inversely, the G1 fraction of the cell cycle in the PC12 cells showed an obvious reduction after the CoCl_2_ exposure, which decreased by 33.19% compared with the untreated control. It is worth noting that the CoCl_2_-stimulated cell cycle arrest was inhibited by the SI treatment. The SI treatments at 50 and 75 μg/mL decreased the cell cycle’s S fraction by 13.36% and 27.79%, respectively, compared with the hypoxic injury group. Meanwhile, the G1 fraction of the cell cycle increased by 13.06% and 30.56%, respectively, compared with the CoCl_2_-stimulated control. To investigate the underlying mechanism, we further analyzed the expression levels of the key proteins cyclin A and p21 involved in the cell cycle. As shown in Figure 3G, the CoCl_2_ exposure significantly down-regulated the cyclin A protein level and up-regulated the p21 protein level compared with the untreated control. However, the SI treatment significantly increased the expression level of the cyclin A protein and decreased the expression level of the p21 protein during CoCl_2_ exposure.

### 3.4. Inhibition of Cell Apoptosis by SIs

The PC12 cell apoptosis rate was monitored using Annexin V–FITC and PI (Figure 3E,F). Apoptotic cells were distributed in two quadrants composed of Q3 (early apoptosis) and Q2 (late apoptosis). The total apoptosis rate (Q2 + Q3) in the CoCl_2_-treated cells was 8.79 times that in the untreated cells, indicating that the CoCl_2_-stimulated hypoxic damage could induce PC12 cell apoptosis. However, this insult in PC12 cell survival was attenuated by SIs, which effectively decreased the total apoptosis rate from 28.29% to 6.11% when the treatment dose of SIs was 75 μg/mL. To investigate the protective mechanism of SIs, the cell apoptosis pathway-associated proteins Bax and Bcl-xL were further examined in the present work. As shown in Figure 3H, the CoCl_2_ exposure significantly increased the pro-apoptotic Bax protein level and decreased the anti-apoptotic Bcl-xL protein level compared with the untreated control. However, the SI treatment significantly increased the expression level of the Bcl-xL protein and decreased the expression level of the Bax protein during CoCl_2_ exposure.

### 3.5. Facilitation of Nrf2 Activation by SIs

**Figure 4:**
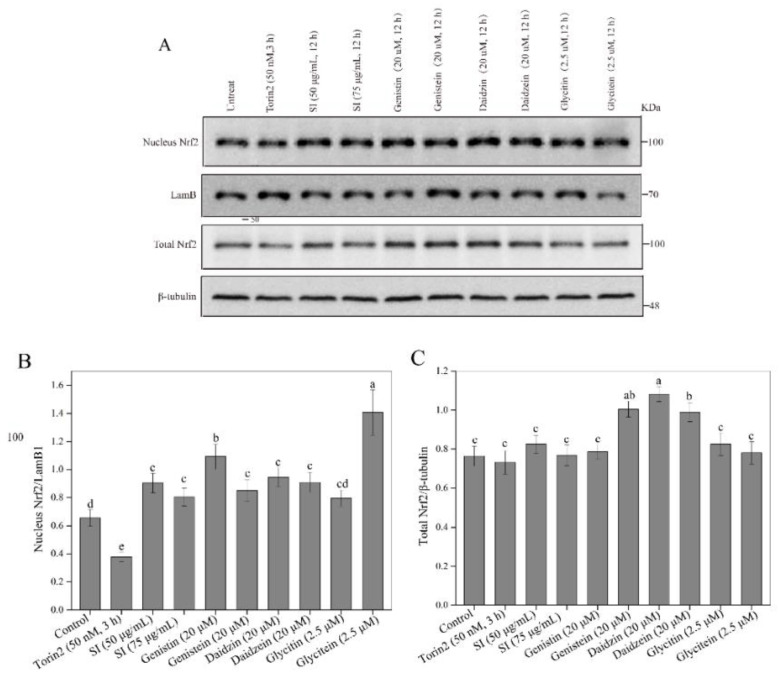
Effects of isoflavones on Nrf2 protein activation. (**A**) Western blot analysis of Nrf2 protein. (**B**) The relative expression level of nuclear Nrf2 protein. (**C**) The relative expression level of total Nrf2 protein. Means with different small letters were significantly different (*p* < 0.05).

**Figure 5:**
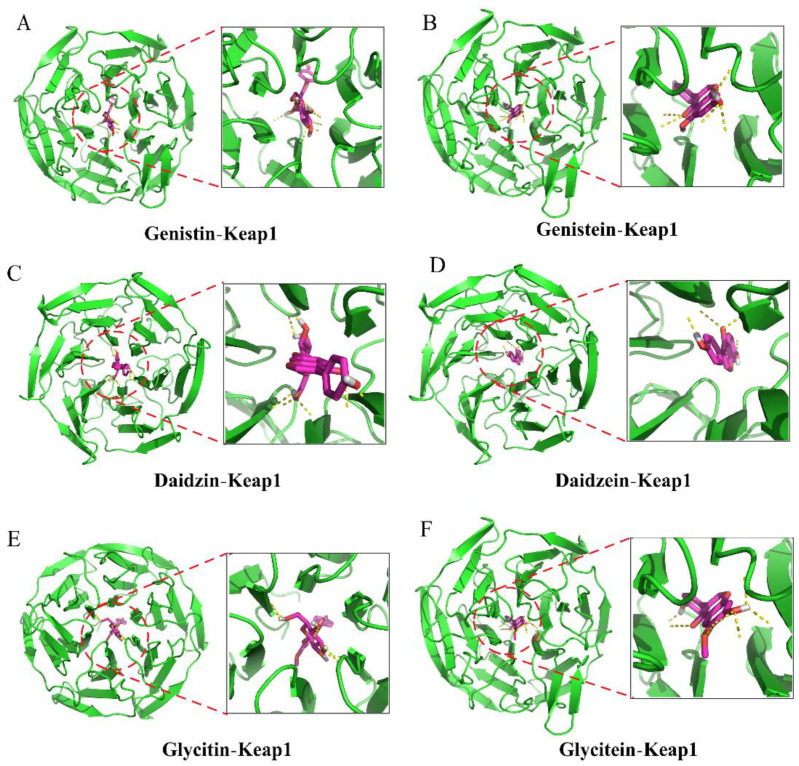
Molecular docking analysis of the interactions between isoflavones and Keap1 protein (3D diagram). (**A**) Genistin bound to keap1. (**B**) Genistein bound to keap1. (**C**) Daidzin bound to keap1. (**D**) Daidzein bound to keap1. (**E**) Glycitin bound to keap1. (**F**) Glycitein bound to keap1.

**Figure 6:**
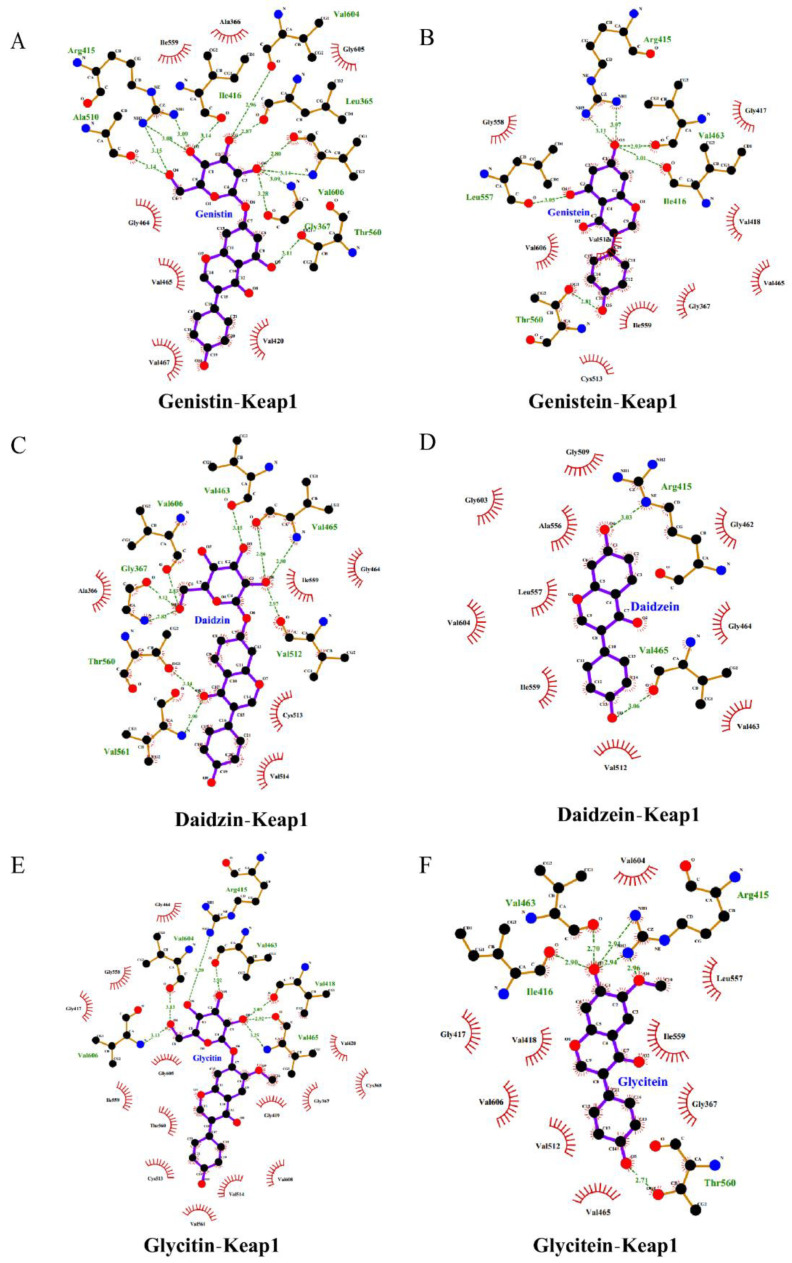
Molecular docking analysis of the interactions between isoflavones and Keap1 protein (2D diagram). (**A**) Genistin bound to keap1. (**B**) Genistein bound to keap1. (**C**) Daidzin bound to keap1. (**D**) Daidzein bound to keap1. (**E**) Glycitin bound to keap1. (**F**) Glycitein bound to keap1.

The Nrf2-Kelch-like ECH-associated protein 1 (Keap1) pathway is a crucial signaling pathway involved in anti-oxidative stress, which can effectively enhance the cellular antioxidant capacity [12]. Hence, we further investigated the effects of isoflavones on the expression level of the Nrf2 protein in PC12 cells. As shown in Figure 4A–C, the SI treatments at 50 and 75 μg/mL did not induce statistical variation in the total Nrf2 level. However, an apparent increase in the nuclear Nrf2 level was observed in SI-treated cells, indicating that SIs promoted the translocation of Nrf2 from the cytoplasm to the nucleus in the PC12 cells. Furthermore, the effects of six isoflavone monomers on the Nrf2 level were also investigated in this study. Genistin, glycitin, and glycitein showed a similar effect to SIs on the total Nrf2 level in PC12 cells, whereas genistein, daidzin, and daidzein significantly (*p* < 0.05) up-regulated the total Nrf2 expression compared to the control. Meanwhile, the nucleus Nrf2 levels were obviously increased after all isoflavone monomer treatments except glycitin compared with control. Regarding the underlying mechanism of isoflavones on the activation of the Nrf2 protein, potential attachment interactions between isoflavone monomers and the Keap1 protein were analyzed using the proposed molecular docking model. Isoflavone monomers can bind to the Keap1 protein through hydrogen bonding and hydrophobic interactions (Figure 5A–F and Figure 6A–F, and Table 1), demonstrating that isoflavones show a promising competitive interaction with the Nrf2 protein.

### 3.6. Activation of Autophagy by Isoflavones

**Figure 7:**
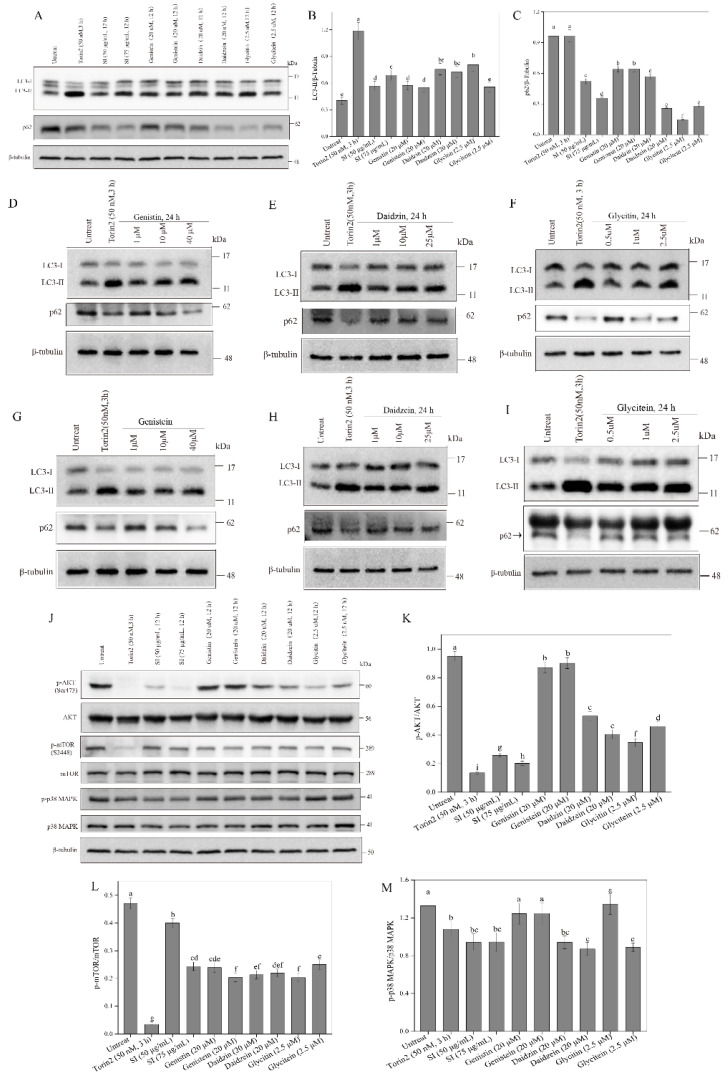
Effects of isoflavones on p38 MAPK pathway and autophagy. (**A**) Effects of isoflavones on autophagy. (**B**) The relative expression level of LC3-II protein in (**A**). (**C**) The relative expression level of p62 protein in (**A**). (**D**) Effects of genistin treatment at different concentrations on autophagy. (**E**) Effects of daidzin treatment at different concentrations on autophagy. (**F**) Effects of glycitin treatment at different concentrations on autophagy. (**G**) Effects of genistein treatment at different concentrations on autophagy. (**H**) Effects of daidzein treatment at different concentrations on autophagy. (**I**) Effects of glycitein treatment at different concentrations on autophagy. (**J**) Western blot analysis of mTOR-AKT and p38 MAPK pathway. (**K**) The phosphorylation of AKT protein. (**L**) The phosphorylation of mTOR protein. (**M**) The phosphorylation of p38 MAPK. Means with different small letters were significantly different (*p* < 0.05).

Evidence shows that autophagy is crucial in chemical hypoxic and oxidant injuries for cell survival [17]. To study the effects of isoflavones on autophagy, the expression levels of the autophagy marker proteins LC3-II and p62 were checked using a Western blot analysis. The autophagy activator Torin2 was employed as a positive control (Figure 7A). The expression level of the LC3-II protein in the PC12 cells was significantly increased by SIs and isoflavone monomers (Figure 7B). For example, the LC3-II level in the 75 μg/mL SI-treated cells was 1.71 times that in the control. The p62 level in PC12 cells showed a significant decrease after the SI treatment (Figure 7C). The expression level of the p62 protein in the 75 μg/mL SI-treated cells was only 36.86% of that in the control. A similar effect of isoflavone monomers was also observed in Figure 7A–C. The results indicated that isoflavones could significantly induce autophagy in PC12 cells.

In the present work, we also used a typical cell model for autophagy, U_2_OS cells, to further study the influence of isoflavones on autophagy. First, the cytotoxicity of isoflavone monomers was investigated in a safe dose. The results are shown in Figure S2. Daidzin, daidzein, glycitin, and glycitein were non-toxic to PC12 cells below 80 μM, 60 μM, 20 μM, and 5 μM, respectively. Genistin and genistein showed no cytotoxicity to PC12 cells even at a high dose of 80 μM. Therefore, safe doses of the isoflavone monomers for U_2_OS cells were used in the following study. As shown in Figure 7D–I, significant increases in the expression levels of the LC3-II protein and significant decreases in the expression levels of the p62 protein were observed after all isoflavone monomer treatments. The results indicated that isoflavones could activate autophagy in a dose-dependent way in U_2_OS cells.

Autophagy is modulated by the AKT–mTOR pathway [18]. Therefore, we further studied the effects of isoflavones on the phosphorylation of AKT and mTOR proteins. As shown in Figure 7J–L, the SI treatment and all the six monomer treatments down-regulated the level of the p-mTOR protein. For example, the level of p-mTOR/mTOR apparently decreased by 48.2% compared with the control when the SI concentration was 75 μg/mL. In addition, the SIs and isoflavone monomers, including daidzein, daidzin, glycitin, and glycitein, also significantly (*p* < 0.05) down-regulated the level of the p-AKT protein. However, genistin and genistein showed no effect on the phosphorylation of AKT. All the results indicated that isoflavones could induce autophagy by inhibiting the AKT–mTOR pathway’s activation.

### 3.7. Inhibition of p38 MAPK Signaling Pathway by Isoflavones

Several studies have demonstrated that apoptosis occurs after the p38 MAPK-dependent activation of the pro-apoptotic protein Bax, and subsequently, cytochrome c releases into the cytosol, activating the caspase cascade under cellular stress [14]. Therefore, the current study examined the effects of isoflavones on the phosphorylation of p38 MAPK. From Figure 7J,M, we can see that the SI treatments at 50 and 75 μg/mL significantly (*p* < 0.05) inhibited the phosphorylation of p38 MAPK compared with the control. A similar result was also observed for the isoflavone monomers, including daidzin, daidzein, and glycitein, wherein the phosphorylation of p38 MAPK respectively decreased by 29.07%, 34.26%, and 33.06%, respectively. However, genistin, genistein, and glycitin showed no effect on the phosphorylation of p38 MAPK, indicating the different biological activities of isoflavone monomers that are possibly due to their differences in structure.

## 4. Discussion

Isoflavones are important polyphenols primarily derived from soybeans and their products. The most outstanding bioactivity of isoflavones is their antioxidant ability due to the aromatic phenolic ring occurring in their structure, which displays free radical-scavenging capacity [19]. Isoflavones have many health-promoting effects in preventing chronic diseases, such as cancers, cardiovascular diseases, and diabetes. In recent years, research on brain disorders has been promoted because of its potential to extend the human lifespan [20]. Brain hypoxia, one of the critical complications caused by impaired brain metabolism, induces a series of pathological responses, especially oxidative stress, in brain functions [1]. In the present study, severe oxidative stress accompanied by a large amount of ROS production was observed using DCFH-DA in the CoCl_2_-induced hypoxic injury model of neural-like PC12 cells. The overproduction of ROS can oxidize proteins, lipids, and DNA; affect mitochondrial function; activate caspase-3; and promote neuronal apoptosis, thereby causing the degeneration of neuronal cells and cognitive performance [20]. Hence, a significant increase in the total apoptosis rate and an obvious S phase cell cycle arrest was observed after CoCl_2_ exposure, thereby resulting in an apparent reduction in the viability of the PC12 cells, which was in line with the findings of Zhang et al. [16]. Jung et al. also found that exposure to CoCl_2_ induced the formation of ROS and caused cell death with the appearance of apoptotic morphology and DNA fragmentation [21]. However, these unfavorable conditions induced by CoCl_2_ were effectively improved by isoflavone treatment, which significantly attenuated the ROS production and cell apoptosis and increased cell viability. As reported by Qian et al., the isoflavone genistein could protect cerebral ischemia mice against oxidative injury by inhibiting ROS production and mitochondria-dependent apoptosis pathways [22]. A report by Levites et al. also indicated that the oxidative damage-induced apoptosis of SH-SY5Y cells derived from human neuroblastoma was markedly repressed by phenolic compounds via the regulation of genes involved in cell survival/the cell cycle [23]. Hence, the underlying protective mechanisms of isoflavones were studied by evaluating key proteins, such as Bax, Bcl-xL, cyclin A, and p21, in the signal pathway involved in cell apoptosis and the cell cycle [24]. Our results suggest that isoflavones protected PC12 cells against CoCl_2_-stimulated cell apoptosis by improving the anti-apoptotic protein Bcl-xL’s expression and suppressing the pro-apoptotic protein Bax’s expression. Meanwhile, isoflavone treatment apparently repressed the cell cycle arrest via an increase in the cyclin A level and a decrease in the p21 level. Furthermore, the cell response to hypoxia was mainly regulated by HIF-1α, which is the active subunit of HIF-1. The severity of hypoxia determines whether cells become apoptotic or adapt to the hypoxia and survive [25]. In this study, treatment with 800 μM CoCl_2_ for 12 h significantly increased the expression of HIF-1α and resulted in significant cell apoptosis, indicating that a chronic or sustained hypoxia in the PC12 cells was induced by the CoCl_2_. Tong et al. also found that HIF-1α was associated with hypoxia-induced apoptosis. However, the increased HIF-1α was inhibited by SIs in the present study [26].

The reported evidence indicates that phenolic compounds, especially isoflavones, can protect nerve cells by activating the intracellular antioxidant system to scavenge ROS [16,27]. Nrf2 is a key regulator in the intracellular antioxidant system responding to oxidative stress. The translocation of Nrf2 from the cytoplasm to the nucleus, and then the binding to ARE, can induce the downstream gene expression of a series of antioxidant enzymes and phase II detoxification enzymes, such as heme oxidase-1 (HO-1), superoxide dismutase (SOD), and glutathion peroxidase (GPx), which play an important role in antioxidant activities, anti-apoptosis, and cell survival [12]. In our previous work, enhanced antioxidant enzyme activities, including catalase (CAT), SOD, and GPx, were observed in PC12 cells treated with an isoflavone-rich extract [24,28]. In the present study, isoflavones could increase the level of nuclear Nrf2 protein, suggesting that isoflavones effectively activate the Nrf2 pathway. Under normal physiological conditions, Keap1 forms a complex with the Nrf2 protein in the cytoplasm, holding its stabilization [11]. Increasing evidence has shown that some natural phytochemicals can bind with the structural domain of the Keap1 protein and exhibit a promising competitive interaction with the Nrf2 protein, thus resulting in its translocation to the nucleus [29]. The molecular docking analysis of the structure–activity relationship between six isoflavone monomers and the Keap1 protein indicated that isoflavone monomers could bind to the Keap1 protein through hydrogen bonding and hydrophobic interactions, thus forming complexes with the Keap1 protein and exhibiting a competitive interaction with the Nrf2 protein, which further confirms the activation of the Nrf2-Keap1/antioxidant response element (ARE) pathway by isoflavones. A previous docking analysis of different natural compounds such as anthocyanins, flavanols, chalcones, and others with the Keap1 protein aligned with our results [29]. In addition, Ji et al. demonstrated that the expression of the HIF-1α gene is involved in the promotion of Nrf2 in C2C12 myoblasts during acute hypoxia [30]. However, Baba et al. found that an increased HIF-1α level and a decreased Nrf2 level were observed in Hep3B, HEK293, and HeLa cells during hypoxia [31]. All the results above indicate that the interaction between Nrf2 and HIF-1 is likely dependent on the cell type and the injury stimulus severity.

The p38 MAPK-dependent pathway can activate the expression of the pro-apoptotic Bax protein, promote the release of cytochrome c into the cytosol, and cause a caspase cascade under cellular stress, thus resulting in cell apoptosis [32]. Our results demonstrate that isoflavones can inhibit the phosphorylation of p38 MAPK and thus block the activation of the MAPK pathway, which is in line with the changes in the Bax and Bcl-xL proteins described above. Ding et al. demonstrated that genistein and folic acid showed neuroprotective effects in β-amyloid peptide-treated neurons by increasing cell viability, decreasing the Ca^2+^ concentration, reducing ROS generation, and inhibiting the expression of p38-MAPK Mrna [33]. Xia et al. also found that dietary polyphenol from rapeseed oil could repress the expression level of caspase-3 and reduce the ratio of Bax/Bcl-2 by inhibiting the phosphorylation of p38 MAPK in hydrogen peroxide-stimulated apoptosis [34]. However, Sánchez et al. reported that genistein selectively enhanced the apoptosis induced by arsenic trioxide in human leukemia cells via ROS formation and the activation of ROS-induced protein kinases (p38 MAPK) [35]. The results above indicate that the effects of isoflavones on the phosphorylation of p38 MAPK might depend on the types of the used models.

**Figure 8:**
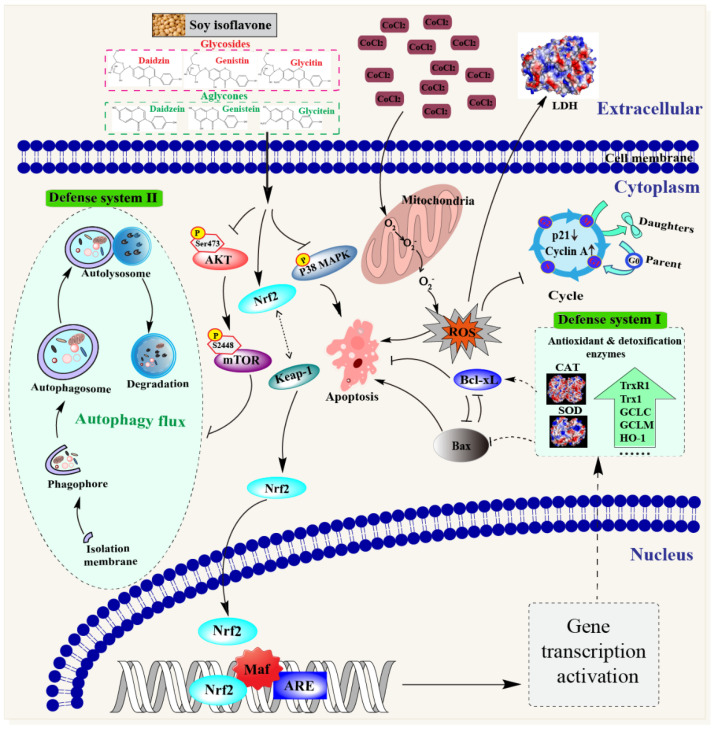
Schematic diagram of the potential mechanisms of the neuroprotective effect of soybean isoflavones.

Autophagy, an important cell survival mechanism, can attenuate hypoxia- and oxidant-induced injuries by the degradation of misfolded proteins and damaged mitochondria [36]. A significant increase in the LC3-II level and decrease in p62 in isoflavone-treated cells indicates that isoflavones could enhance the degradation of the damaged components in PC12 cells through autophagy activation. Similar results were also obtained from several previous reports. Li et al. found that isoflavones inhibited the atrazine-induced apoptosis of SH-SY5Y cells by improving BEX2-dependent autophagy [37]. Cheng, Kao, and Lee also indicated that ferulic acid exhibited anti-apoptotic activity against ischemic damage by inducing HSP70/Bcl-2- and HSP70/autophagy-mediated signaling in rats [38]. The activation of autophagy is modulated by the AKT–mTOR signaling pathway, wherein the mTOR protein suppresses autophagy and this suppressive action is mediated by AKT signaling [18]. We found that the SI treatment and its monomers could decrease the phosphorylation of AKT and mTOR proteins, indicating that isoflavones induced autophagy via inhibiting the activation of the AKT–mTOR pathway. Consequently, the neuroprotective effects of isoflavones might be partly owed to the induced autophagy through the modulation of the AKT–mTOR pathway, which enhanced the ability of PC12 cells to remove harmful metabolic waste in hypoxic damage. As summarized in Figure 8, isoflavones might exhibit protective effects on PC12 cells in vitro via the following pathways: (1) triggering the antioxidant mechanism to scavenge ROS through activating the Nrf2–Keap1 pathway; (2) facilitating cell cycle arrest by increasing the expression of the cyclin A protein and decreasing the expression of the p21 protein; (3) blocking apoptosis through the suppression of the p38 MAPK pathway; and (4) inducing autophagy to eliminate the damaged cellular components through the inhibition of the AKT–mTOR pathway.

## 5. Conclusions

The current study indicates that isoflavones exert protective effects on neuronal PC12 cells against hypoxic damage. Isoflavones decreased the ROS production by activating the Nrf2–Keap1 pathway, reduced cell apoptosis through the suppression of the p38 MAPK pathway, and induced autophagy to remove the damaged cellular components by inhibiting the AKT–mTOR pathway. Overall, our findings indicate that isoflavones have the potential to be developed as a functional additive to prevent neuron damage.

## Figures and Tables

**Table 1:** Details of the docked complex of Keap1 with isoflavones.

Molecule	Docking Energy (kcal/mol)	Hydrogen-Bonding Interactions	Hydrogen Bond Distance	Key Hydrophobic Interactions
Daidzin	−10.7	Val561	2.90	Ala366, Gly513, Val465, Val514, Ile559, Gly464
		Thr560	3.14	
		Gly367	3.13, 2.82	
		Val606	2.83	
		Val463	3.15	
		Val465	2.86, 2.80	
		Val512	2.97	
Glycitin	−10.4	Val606	3.13	Gly464, Gly558, Gly417, Gly605, Ile559, Thr560, Cys513, Val561, Val514, Val608, Gly419, Gly367, Cys368, Val420
		Val604	3.13	
		Arg415	3.20	
		Val463	2.92	
		Val418	3.03	
		Val465	2.92, 3.25	
Genistin	−10.7	Ala510	3.14	Ile559, Ala366, Gly605, Gly464, Val465, Gly467, Val420
		Arg415	3.15, 3.08, 3.09	
		Ile416	3.14	
		Val604	2.96	
		Leu365	2.87	
		Val606	2.80, 3.14	
		Gly367	3.28, 3.09	
		Thr560	3.11	
Daidzein	−9.7	Arg415	3.03	Gly509, Gly603, Ala556, Leu557, Val604, Ile559, Val512, Val463, Gly464, Gly462
		Val465	3.06	
Glycitein	−9.3	Ile416	2.90	Val604, Gly417, Val418, Val606, Val512, Val465, Leu557, Ile559, Gly367
		Arg415	2.91, 2.94, 2.96	
		Val463	2.70	
		Thr560	2.71	
Genistein	−9.2	Arg415	3.11, 3.07	Gly558, Val606, Cys513, Ile559, Gly367, Val465, Gly417, Val418
		Leu557	3.05	
		Val463	2.93	
		Ile416	3.01	
		Thr560	2.81	

## Data Availability

The data presented in this study are available from the corresponding author upon request.

## Supplementary Materials

The following supporting information can be downloaded at: https://www.mdpi.com/article/10.3390/antiox11102037/s1, Figure S1 (The protective potency of six isoflavone monomers on PC12 cell viability under hypoxic injury) and S2 (Cytotoxicity of isoflavone monomers for U 2 OS cells).
