# Supplementary material for: Soy Isoflavones Protect Neuronal PC12 Cells against Hypoxic Damage through Nrf2 Activation and Suppression of p38 MAPK and AKT–mTOR Pathways
Source: Antioxidants (Basel). 2022 Oct 16;11(10):2037. doi: 10.3390/antiox11102037 (PMC9598610; doi:10.3390/antiox11102037)
Supplement: Supplementary file 1 [file antioxidants-11-02037-s001.zip › antioxidants-1905651-supplementary.pdf]

Supplementary Figure legends

Figure S1 The protective potency of six isoflavone monomers on PC12 cell viability under hypoxic injury

Figure S2 Cytotoxicity of isoflavone monomers for U_2OS cells. (A) Daidzin and daidzein. (B) Genisitin and genistein. (D) Glycitin and glycitein.

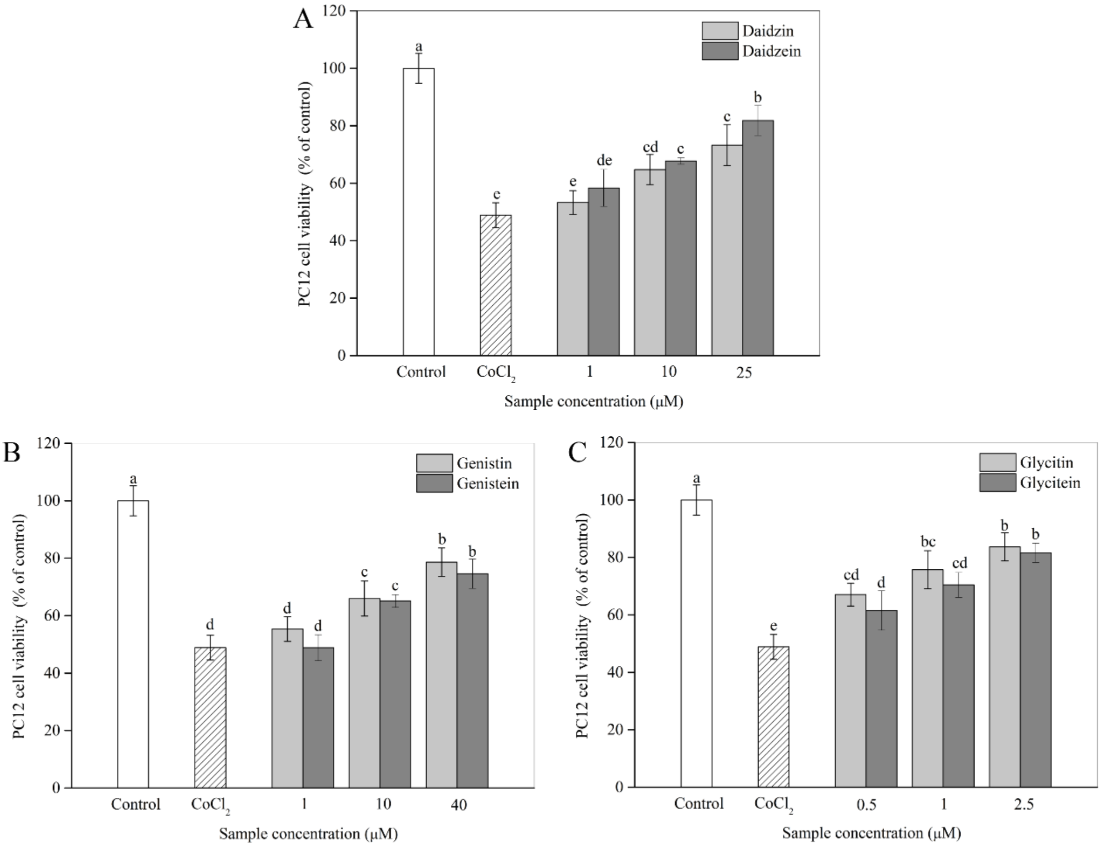

Figure S1

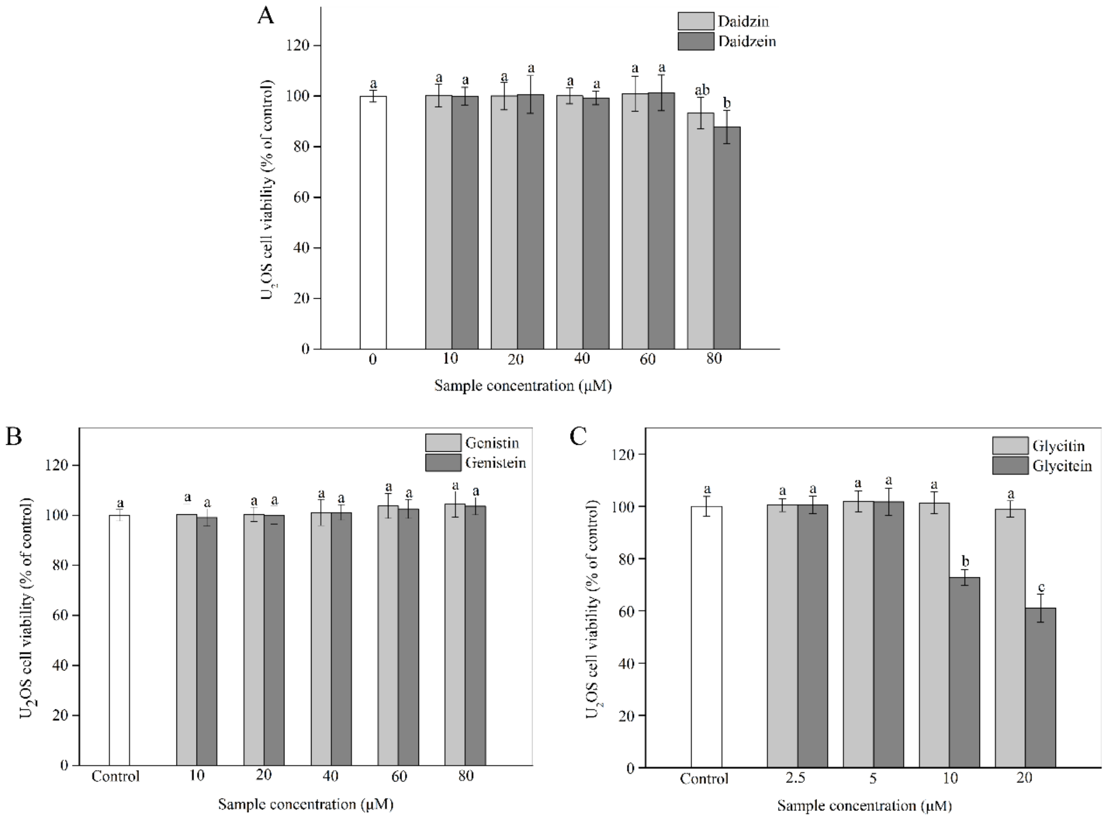
A
Daidzin
Daidzein
U2OS cell viability (% of control)
Sample concentration (μM)
B
Genistin
Genistein
U2OS cell viability (% of control)
Sample concentration (μM)
C
Glycitin
Glycitein
U2OS cell viability (% of control)
Sample concentration (μM)

Figure S2
